# Supplementary material for: MetaRibo-Seq measures translation in microbiomes
Source: Nat Commun. 2020 Jun 29;11:3268. doi: 10.1038/s41467-020-17081-z (PMC7324362; doi:10.1038/s41467-020-17081-z)
Supplement: Supplementary file 10 — Supplementary Data 7 [file 41467_2020_17081_MOESM10_ESM.zip › File2/Confidence_High_Taxonomy/310577_out.krona.html]

Javascript must be enabled to view this page.

members
magnitude
magnitudeUnassigned
count
unassigned
taxon
rank

310577\_out

2

2
superkingdom
2

1239
phylum
2

186801
class
2

2
order
186802

2
186803
family

genus
1164882
1

1

SRS024087\_contig\_number\_19095
species
467210

genus
177971
1

1

SRS019125\_contig\_number\_595
species
177972
